# Right ventricular mass has better reproducibility in systole than diastole in patients with suspected pulmonary hypertension

**DOI:** 10.1186/1532-429X-17-S1-P174

**Published:** 2015-02-03

**Authors:** Andrew J Swift, Dave Capener, David G Kiely, Jim M Wild

**Affiliations:** Department of cardiovascular Science, University of Sheffield, Sheffield, UK; Sheffield Pulmonary Vascular Disease Unit, Sheffield Teaching Hospitals NHS Trust, Sheffield, UK

## Background

Accurate estimation of right ventricular (RV) measurements is important in the clinical assessment of patients with pulmonary hypertension and has a potential role as an end-point in clinical trials. However, there is no clear agreement as to how RV mass measurements should be performed. RV mass is traditionally measured in diastole even though RV musculature is known to be thicker in systole as compared to diastole. The aim of this study was to determine the reproducibility of RV mass measurements in patients with pulmonary hypertension in both systolic and diastolic phases of the cardiac cycle.

## Methods

MRI scans from consecutive patients with suspected pulmonary hypertension referred to a large volume pulmonary hypertension centre were analysed by two independent observers. Both observers were blinded to the patient's demographic, clinical, and invasive haemodynamic data. The RV epicardial and endocardial borders on each end-diastolic, and end-systolic, short axis slices were traced. As described in previous work the interventricular septum was considered as part of the LV. The product of the sum total of the myocardial slice volumes for each ventricle and the density of myocardium (1.05 g/cm3) gave an estimate of RV mass.The inter-observer and intra-observer agreement of RV mass was assessed using intra-class coefficient (ICC) analysis was performed and Bland-Altman plots were constructed.

## Results

**Figure 1:**
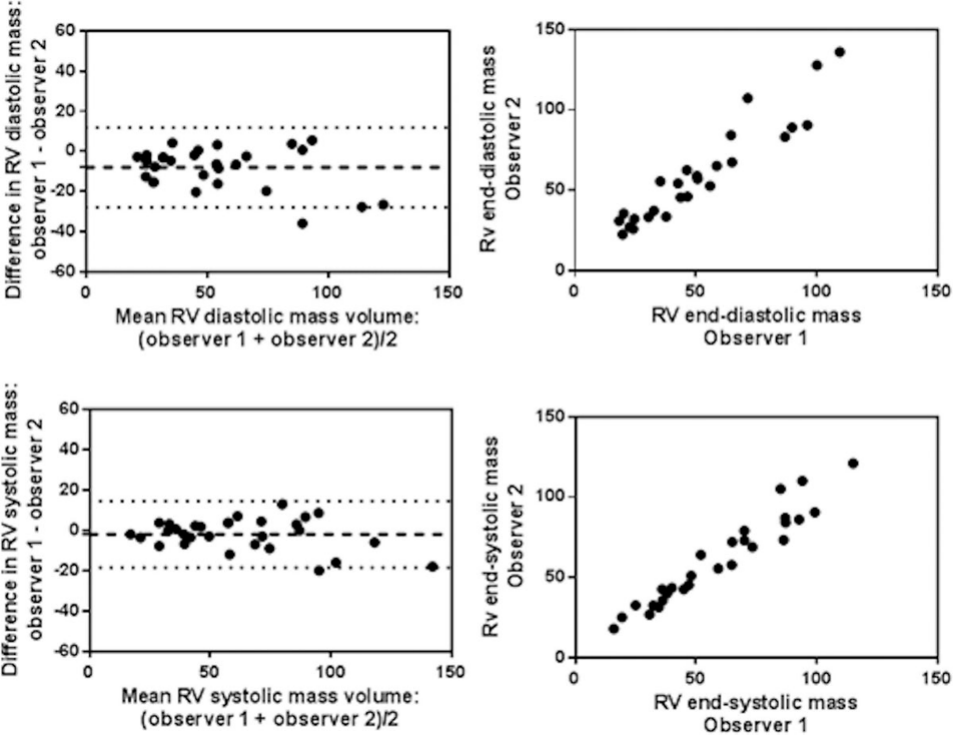
Bland-Altman plots and scatter plots showing the inter-observer variability for RV mass in systole and diastole.

**Figure 2:**
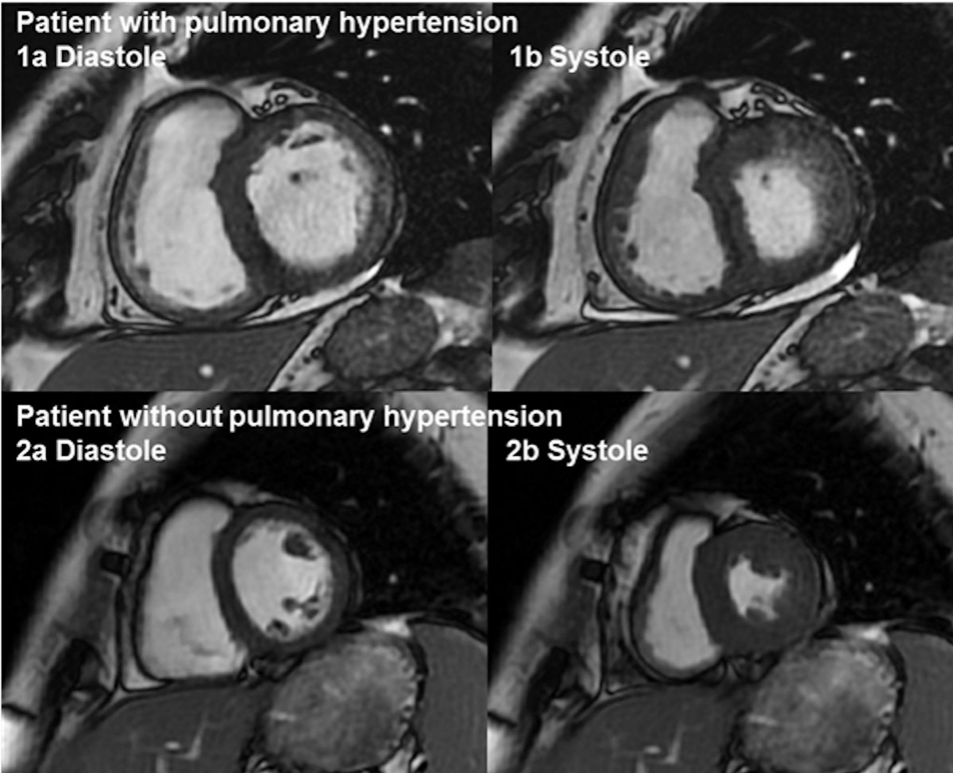
**Showing matched short axis slices in systole and diastole in a patient with and without pulmonary hypertension.** (1) A patient with pulmonary hypertension, image in diastole (a) the RV mass is less conspicuous than shown in systole (b), as a result the RV endocardial border is more easily appreciated in systole compared to diastole. This observation is mirrored in the patient without pulmonary hypertension (2), and likely explains the higher mean systolic RV mass and its higher reproducibility.

30 consecutive patients with suspected pulmonary hypertension were studied by both independent observers. RV mass measured in systole had higher ICC agreement than diastole (0.983 verses 0.947) and showed less bias at Bland-Altman analysis, 1.9g compared to 8.7g, Figure 1. Of note, RV mass measurements were significantly higher when measured in systole than diastole, with a mean difference of 10g (95% confidence interval of 6.6 to 13.2, p<0.0001). Bland Altman analysis shows RV mass measured in systole to be systematically higher than in diastole with a bias of 9.9g, with limits of agreement ranging from -27.4g to 7.6g. Figure 2 illustrates that the RV endocardial border is more easily appreciated in systole compared to diastole in patients with and without pulmonary hypertension.

## Conclusions

RV mass possess a higher reproducibility profile at end-systole than end-diastole in patients with suspected pulmonary hypertension, this finding is of clinical relevance as mass measurements are typically measured in diastole.

## Funding

AJS and JMW received funding from the National Institute for Health Research (NIHR). JMW is also funded by the Engineering and Physical Sciences Research Council (EPSRC). DC receives funding from Bayer Schering.

